# Supplementary material for: Phlag: scalable detection of genomics regions with unexplained phylogenetic heterogeneity
Source: Bioinformatics. 2026 Jul 7;42(Suppl 1):btag273. doi: 10.1093/bioinformatics/btag273 (PMC13340173; doi:10.1093/bioinformatics/btag273)
Supplement: btag273_Supplementary_Data [file btag273_supplementary_data.pdf]

Supplementary Material

Supplementary Figures

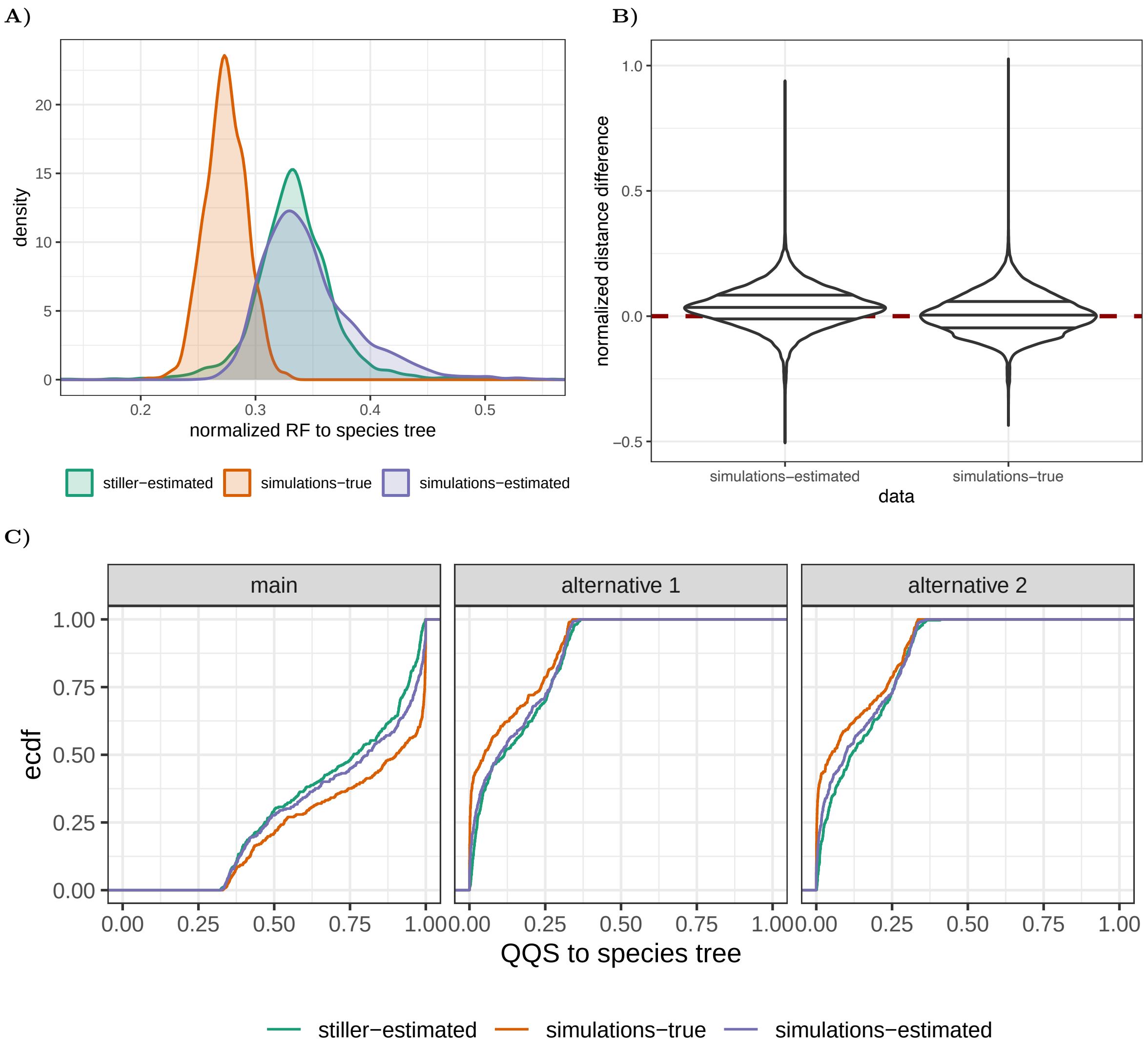

Fig. S1. Summary statistics of the simulated gene trees. We show three sets of gene trees: *i*) stiller-estimated: gene trees estimated by Stiller et al. (2024) from real data; *ii*) simulations-true: "true" simulated genealogies generated using msprime; and *iii*) simulations-estimated: gene trees estimated using IQ-TREE from simulated sequences of 500 bp regions. A) Distribution of normalized RF distances between gene trees and the reference species tree. Note the close match between the simulations-estimated gene trees used in our analyses and the stiller-estimated trees. B) The difference in pairwise distances (in substitution units) between simulated and Stiller et al. (2024) gene trees. We compare the average pairwise distance between a pair of taxa across all gene trees for simulations $\hat{d}$ versus Stiller et al. (2024) gene trees d^*. The distributions are shown over all pairs of taxa. We show $\frac{\hat{d}-d^*}{d^*}$. Distributions are centered around zero, showing that selected substitution rates lead to gene trees that match empirical data. C) Empirical distribution function of Quadripartition Quartet Scores (QQS) of gene trees with respect to the reference species tree. Note the close match between the empirical data (stiller-estimated) and the simulated data (simulations-estimated).

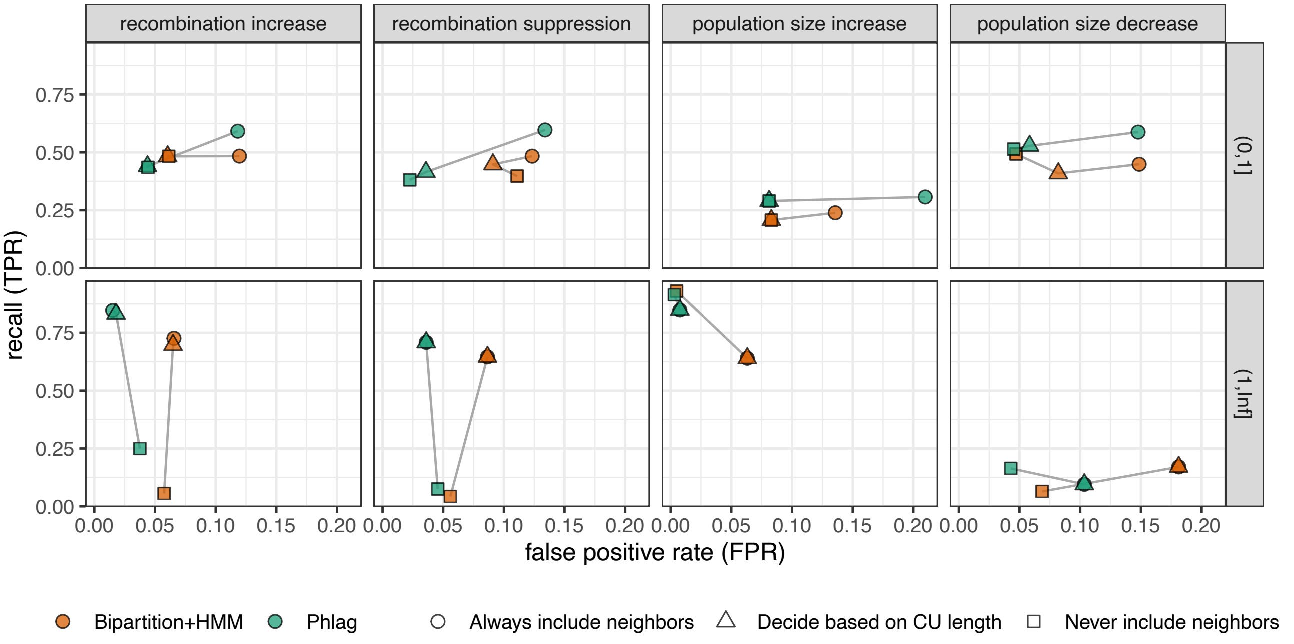

Fig. S2. Demonstrating the impact of expanding $\mathcal{B}'$ by adding neighbor branches of the focal branch. We show the false positive rate (FPR) on the x-axis, and the true positive rate on the y-axis. Shapes indicate the expansion strategy. "Decide based on CU length" strategy adds neighbor branches if the focal branch length estimate is greater than 1 CU. We note that the decision is made based on the estimated branch lengths. We show the true CU branch lenghts in panel rows.

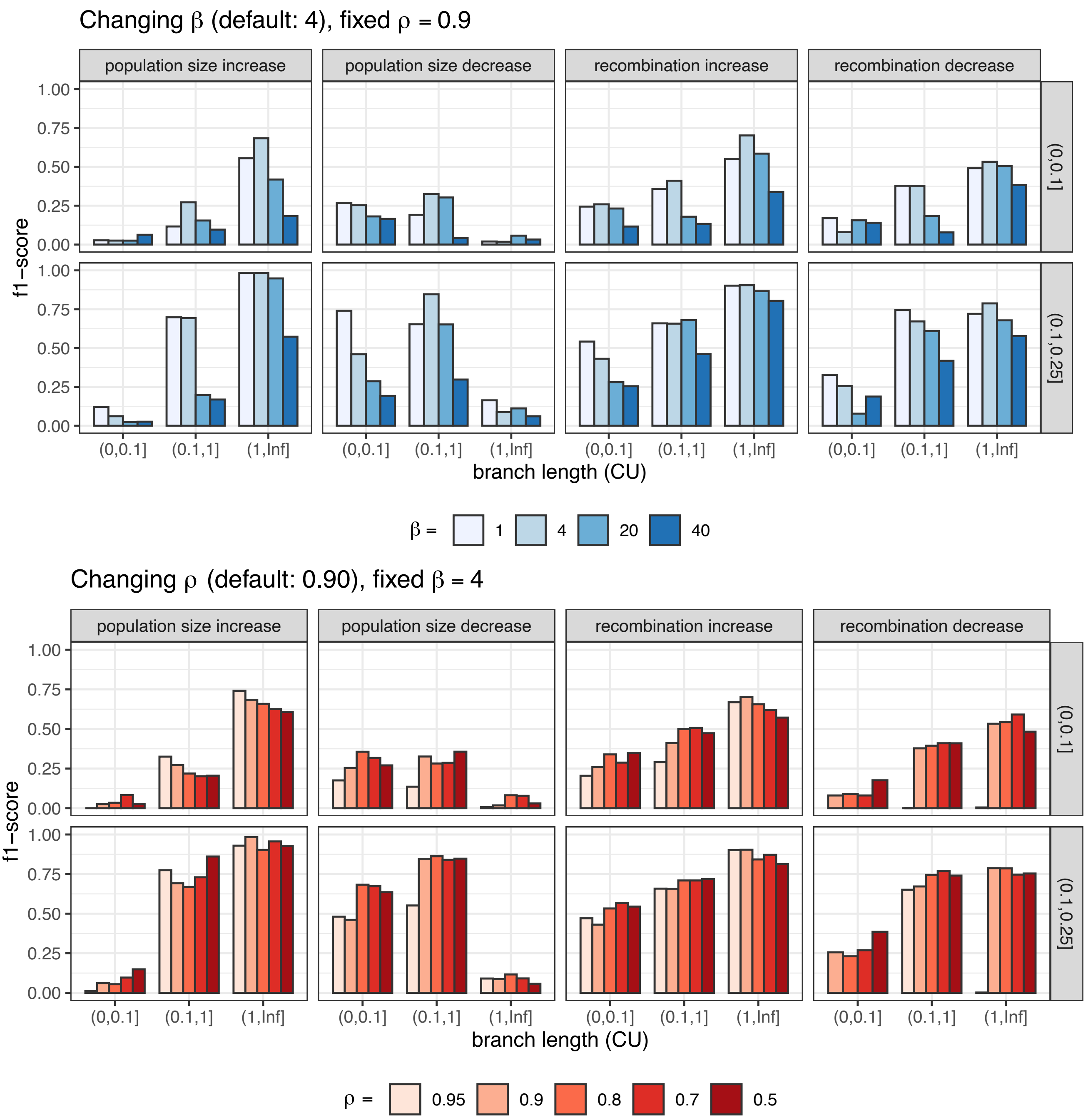

Fig. S3. Showing the impact of varying pior hyperparameters $\beta \in \{1, 4, 20, 40\}$ (top) and $\rho \in \{0.50, 0.70, 0.80, 0.90, 0.95\}$ (bottom) defined in Eq. (1) on the performance shown as the f1-score on the y-axis. Branches are binned based on CU lengths on the x-axis. Panel columns are for different types of alternative states, and panel rows correspond to the portion of affected gene trees.

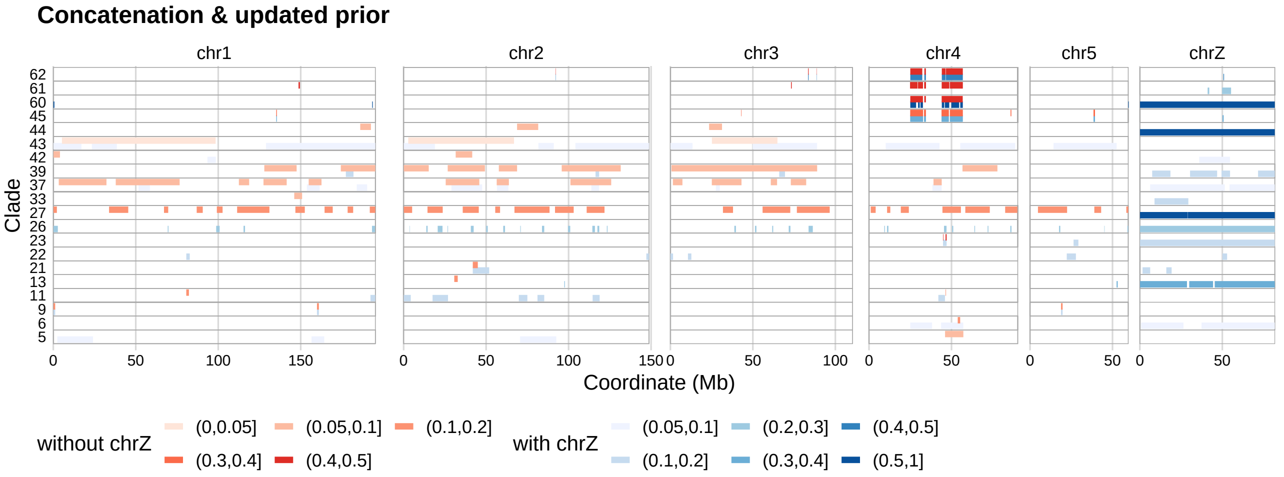

Fig. S4. Phlag analysis of bird macrochromosomes and the Z chromosome. We ran Phlag twice, once with and once without chromosome Z included. Analyses that exclude the Z chromosome are shown with red colors (upper part of each row) and those that include the Z chromosome are shown with blue colors (lower part of each row). The analyses that exclude the Z chromosome are identical to those in Fig. 4. In general, inclusion of the Z chromosome reduced the number of autosomal outliers detected by Phlag, although there are a few exceptions where short autosomal regions were detected when the Z chromosome was included (e.g., branch 11). This pattern can be explained by the observation that our model includes only one alternative state; thus, if the Z chromosomes show a stronger pattern of disagreement with the default MSC model, it can prevent the detection of more subtle deviations. Employing iterations is a potential solution.

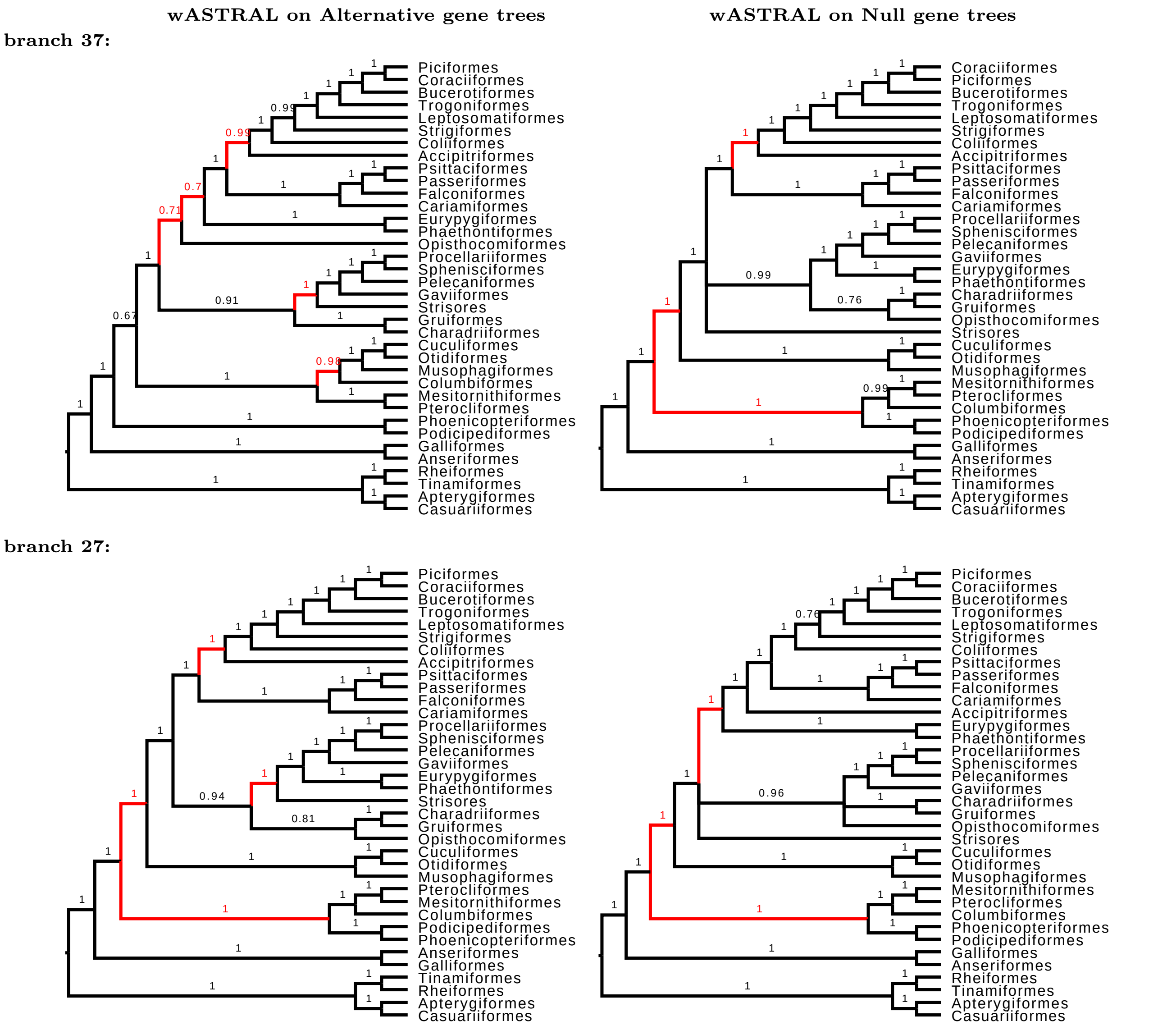

Fig. S5. Trees generated by running wASTRAL on gene trees from the null or alternative regions for branches 37 and 27 of birds. This figure shows all avian orders; the versions in Fig. 4 are reduced to show the focal taxa predicted to change. Note that the species tree includes 363 species; we collapse all the orders in the visualizations. We removed orders outside of Neoaves, which we do not study here.

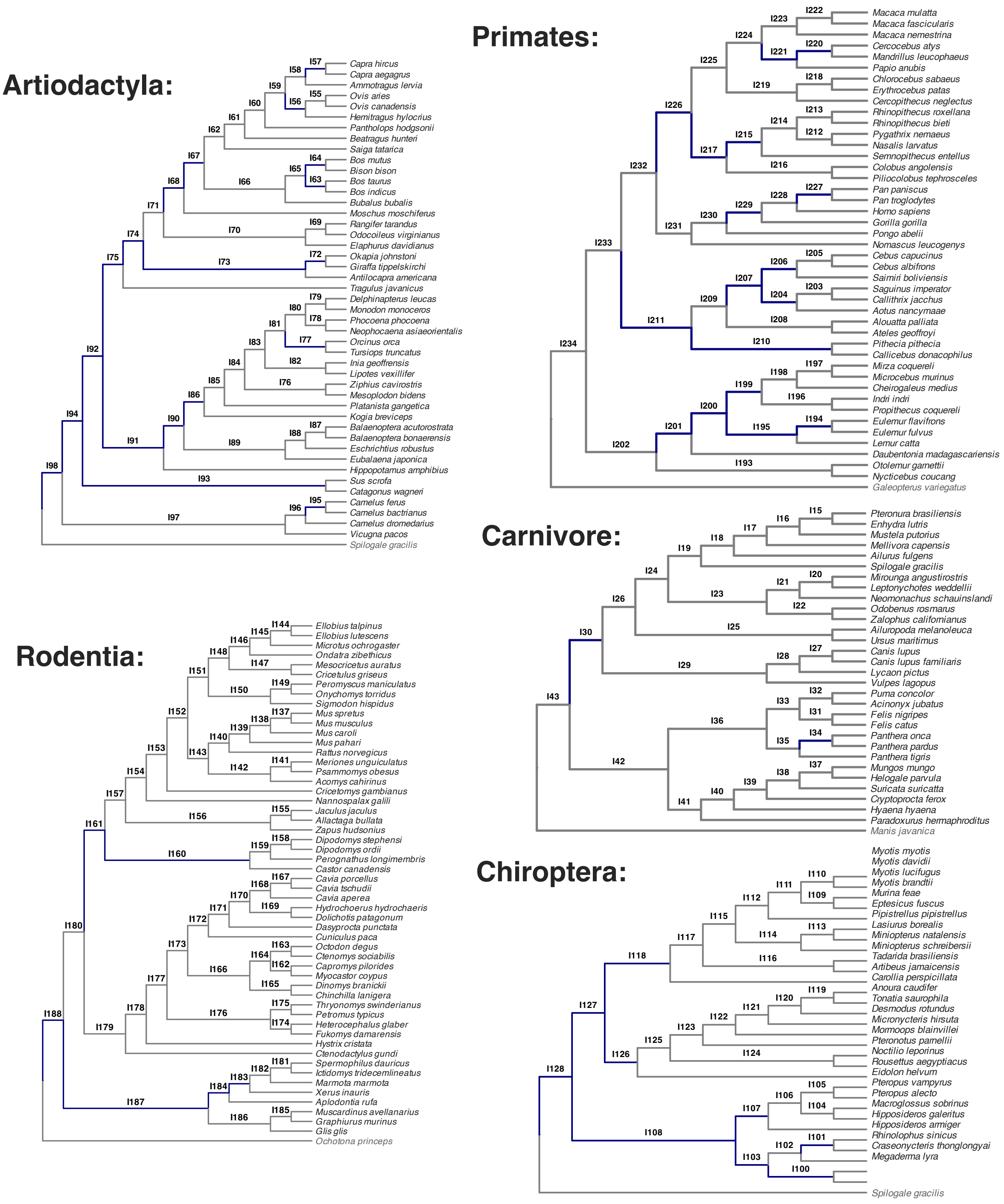

Fig. S6. Showing the subtrees from the five key orders selected from the mammalian phylogeny, namely Artiodactyla, Primates, Rodentia, Carnivora, and Chiroptera. Shown clades correspond to the common ancestor node above the edges with regions identified by Phlag with >0.25 Hellinger distance, highlighted in blue.

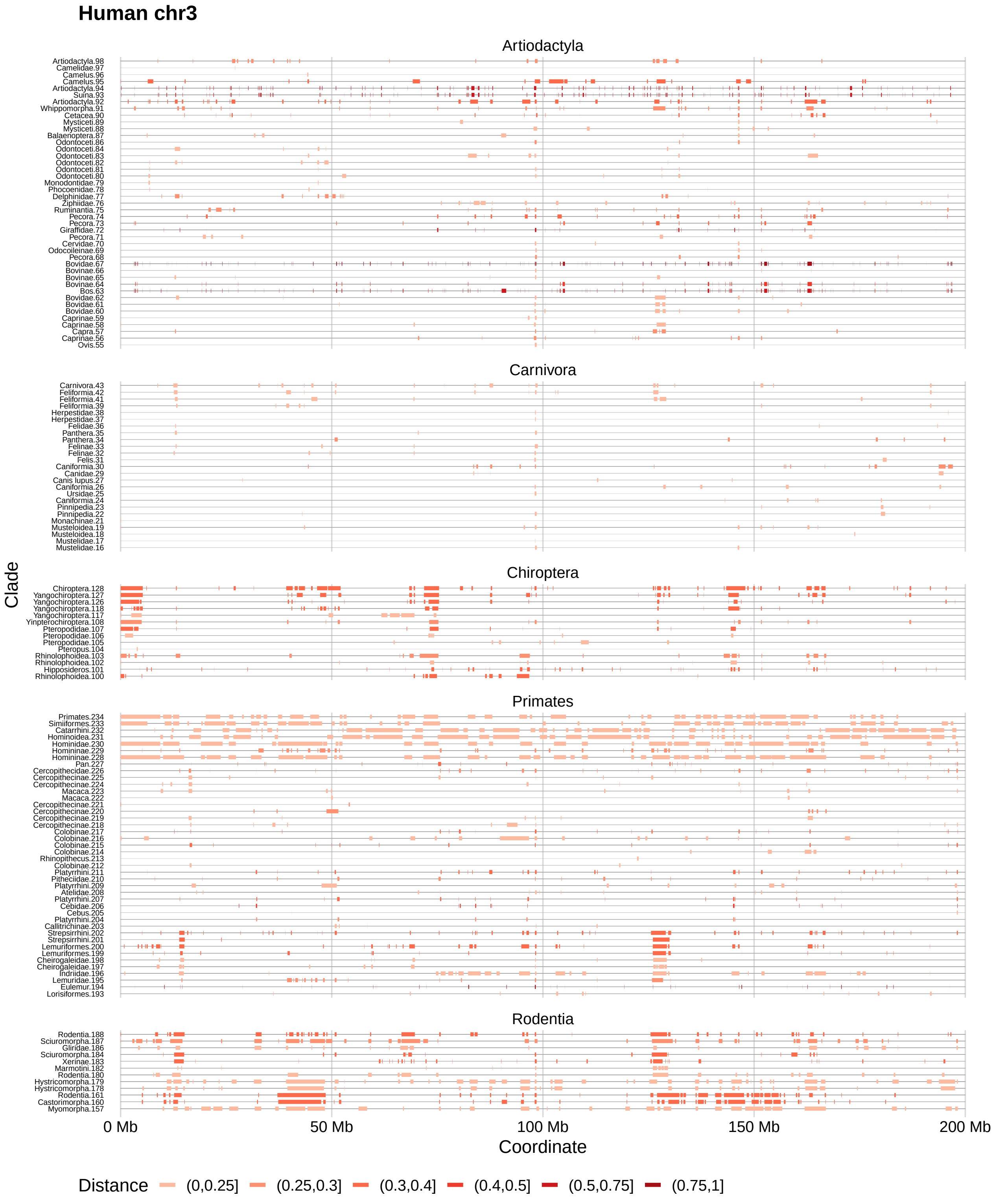

Fig. S7. The complete results of the Phlag analysis on the mammal dataset were obtained using the same hyperparameters as the analysis conducted in the manuscript ($\beta = 100$ and $\rho = 0.95$). Each row corresponds to a branch in the mammal tree. This figure presents results for all individual branches from Carnivora, Chiroptera, Primates, Artiodactyla, and Rodentia. All branches with sufficiently complete data (>90%) are included. In Figure 5, we highlight branches with distances exceeding 0.25. For each branch, we report the lowest common ancestor (LCA) of all taxa in the subtree rooted at the descendant node, along with the order in which this node is visited during the post-order traversal on the y-axis.

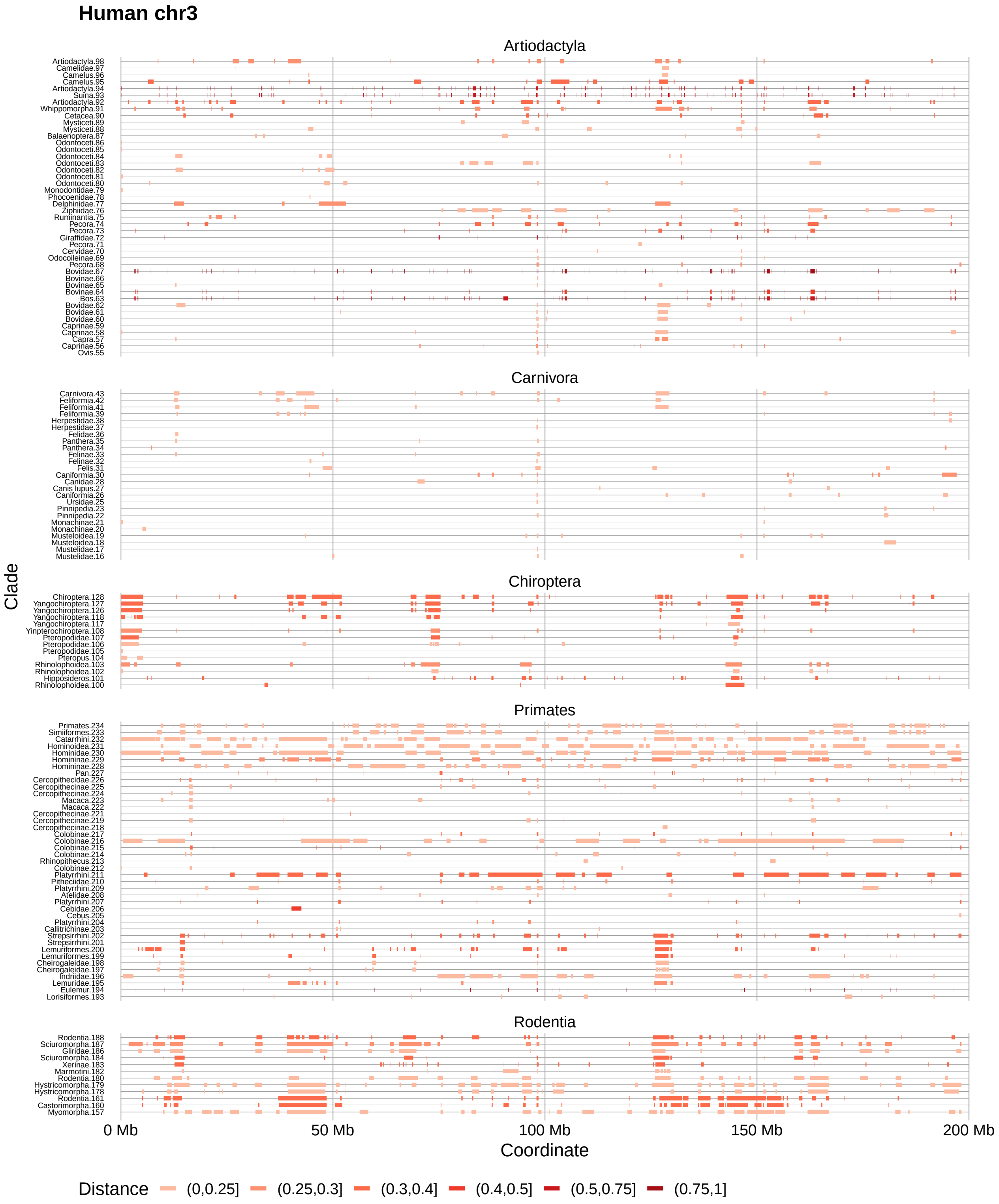

Fig. S8. The complete results of the Phlag analysis on the mammal dataset were obtained by setting $\beta = 50$ and $\rho = 0.95$ to maintain an expected number of transitions per gene tree of $\frac{\beta}{N} \approx 0.0026$, where $N = 19,465$.

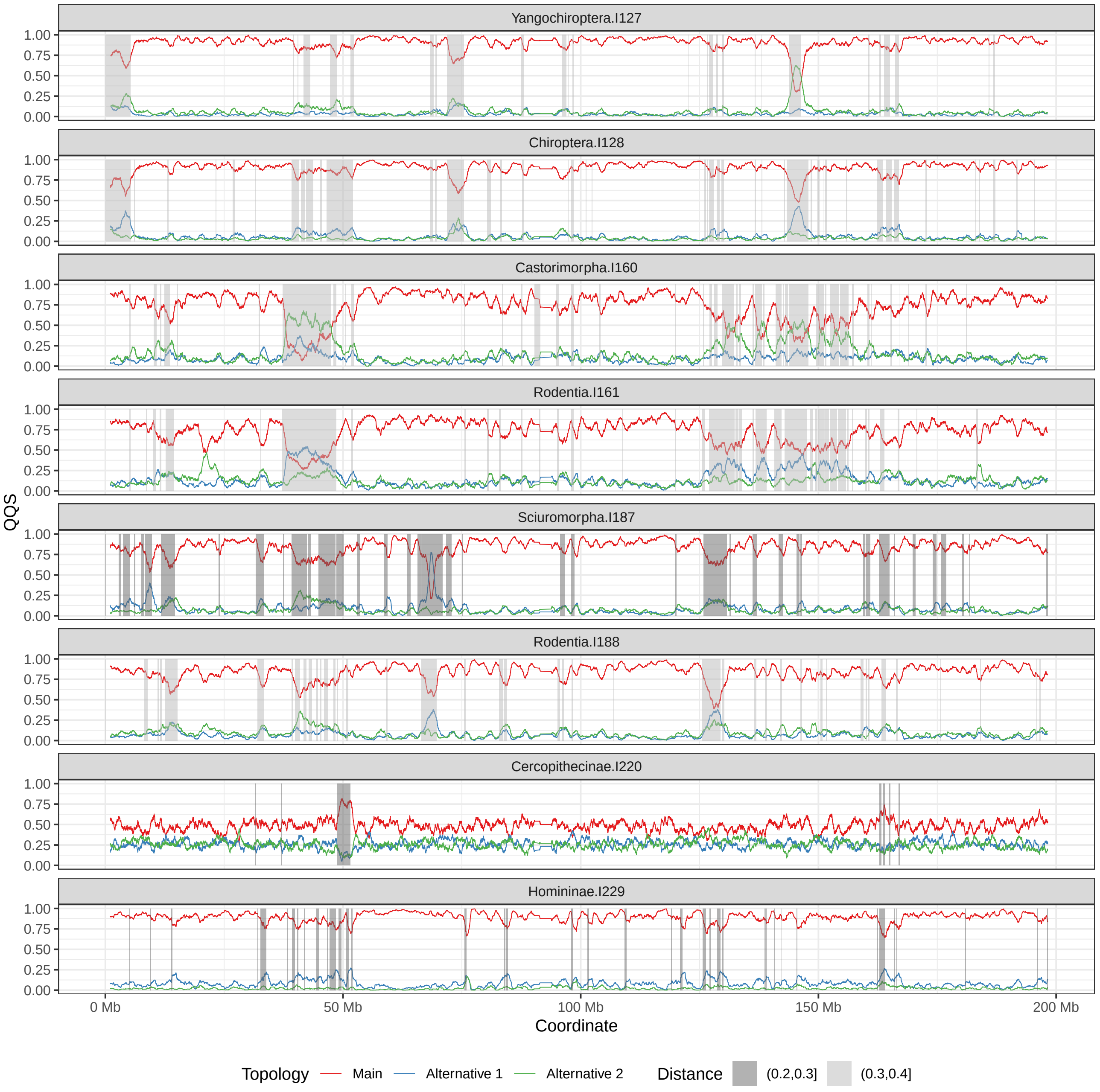

Fig. S9. Moving averages of Quartet Quadripartition Support (QQS) values of three topologies for 8 focal branches from Figure S7. Regions identified by Phlag are highlighted in gray, with shading based on the Hellinger distance between the emission distributions of the two states.

Supplementary Notes

Derivation of the transition matrix priors

We consider a two-state transition matrix

$$P = \begin{pmatrix} p_0 & 1 - p_0 \\ 1 - p_1 & p_1 \end{pmatrix},$$

where $p_0 = p(Z_i = 0 \mid Z_{i-1} = 0)$ and $p_1 = p(Z_i = 1 \mid Z_{i-1} = 1)$. The stationary distribution $\pi^* = [\rho, 1-\rho]$ satisfies $\pi^* P = \pi^*$. Solving for ρ results in

$$\rho = \frac{1 - p_1}{2 - p_0 - p_1}.$$

where ρ is the prior probability of being in the 0 state.

Let N denote the sequence length and let β denote the expected number of subsequences with $Z_i = 1$. Under stationarity, the expected number of $0 \to 1$ transitions is $N\rho(1 - p_0)$. Thus, $N\rho(1 - p_0) = \beta$ yields $p_0 = 1 - \beta/(N\rho)$. Therefore, we end up with

$$p_0 = \frac{N\rho - \beta}{N\rho} \text{ and } p_1 = \frac{\beta - N + N\rho}{N(\rho - 1)}.$$

We place conjugate priors $p_0 \sim \text{Beta}(\alpha_0, \beta)$ and $p_1 \sim \text{Beta}(\alpha_1, \beta)$, with expectations $\mathbb{E}[p_0] = \frac{\alpha_0}{\alpha_0+\beta}$ and $\mathbb{E}[p_1] = \frac{\alpha_1}{\alpha_1+\beta}$. Enforcing the above constraints together with $\alpha_0 + \beta = N\rho$ and $\alpha_1 + \beta = N(1 - \rho)$ gives

$$\alpha_0 = N\rho - \beta \text{ and } \alpha_1 = N(1 - \rho) - \beta.$$

M-step

In the M-step, we update model parameters by maximizing the expected complete log-posterior. Specifically, we solve

$$\underset{e_\emptyset, e_a, P, \pi}{\arg\max}\ \mathbb{E}_{Z \mid Y}\left[\log p(Y, Z \mid e_\emptyset, e_a, P, \pi)\right] + \log p(e_\emptyset \mid \hat{T}) + \log p(P).$$

The emission parameters $e_\emptyset$ and e_a are updated independently across branches by maximizing

$$\sum_{j=1}^{m}\left[\left(\sum_{i=1}^{N}\gamma_i \log p(Y_i^{(j)} \mid e_\emptyset^{(j)})\right) - \lambda\,\mathrm{d}(e_\emptyset^{(j)}, \tilde{e}_\emptyset^{(j)})\right] \text{ and } \sum_{i=1}^{N}(1-\gamma_i)\sum_{j=1}^{m}\log p(Y_i^{(j)} \mid e_a^{(j)}), \text{ respectively.}$$

The resulting updates correspond to maximum a posteriori estimates for $e_\emptyset$ and maximum likelihood estimates for e_a. For instance, when $Y_i^{(j)} \sim \text{Categorical}(e_a^{(j)})$, which is the case for the default topology order emissions, MLE is simply

$$e_a^{(j)}(k) = \frac{\sum_{i=1}^{N} \mathbb{1}_{Y_i^{(j)}=k}(1-\gamma_i)}{\sum_{i=1}^{N}(1-\gamma_i)}. \tag{1}$$

Unlike the maximum likelihood objective of e_a, the objective for $e_\emptyset$ generally admits no closed-form solution due to the distance-based prior term Eq. (3), and hence, the maximization is performed numerically. In particular, we use L-BFGS-B without using the explicit derivative.

The transition probabilities are updated using the expected transition counts as

$$p_0 = \frac{\sum_{i=1}^{N-1}\xi_{i,i+1}(0,0) + \alpha_0 - 1}{\sum_{i=1}^{N-1}\gamma_i + \alpha_0 + \beta - 2} \text{ and } p_1 = \frac{\sum_{i=1}^{N-1}\xi_{i,i+1}(1,1) + \alpha_1 - 1}{\sum_{i=1}^{N-1}(1-\gamma_i) + \alpha_1 + \beta - 2}.$$

The initial distribution π is updated as

$$\pi_1 = \gamma_1 \text{ and } \pi_0 = 1 - \gamma_1.$$

Gene tree simulations

Ancestry simulations: We simulate genealogies under the demographic model of the avian species tree inferred by Stiller *et al.* (Stiller et al., 2024) using Hudson's algorithm implemented in `msprime`. We set the recombination rate to 5×10^{-9} and fix the generation time at 10 years across the entire tree. For computational feasibility, we simulate sequences of length 500Kbp and concatenate 12 independent blocks of 500Kbp to obtain 6Mbp-long sequences. We respect these concatenation boundaries when sampling genes for gene tree estimation to minimize the error introduced. We note that the resulting genealogy sequences produced by `msprime` are ultrametric.

Incorporating varying rates of evolution: We extend the simulations to incorporate variation in rates of evolution by first scaling the rooted species tree branch lengths, measured in numbers of generations, by branch-specific rate multipliers; i.e., branch lengths in substitution units normalized by the average substitution rate. Note that these lengths are obtained empirically on real data. This produces a non-ultrametric species tree, for which we reassign node times by anchoring the tip with the greatest root-to-tip distance at time zero and shifting all other nodes accordingly. Formally, let $h(x)$, $d(x)$, and $t(x)$ denote the height, depth, and time of a species tree node x before scaling, and let $h'(x)$, $d'(x)$, and $t'(x)$ denote the corresponding quantities after scaling. We assign each species tree node a new time $t'(x) = h(\text{root}) - d'(x)$. Since each node in a simulated genealogy belongs to a population corresponding to a species tree node, a genealogy node y sampled at time $t(y)$ within population x is assigned a scaled time

$$t'(y) = t'(x) + r(x)\,(t(y) - t(x))\,,$$

where $r(x)$ is the rate multiplier of the branch connecting x to its parent in the rooted species tree. The effect of the procedure described is to assign a substitution rate to each branch of the species tree and scale each branch of the gene tree by the weighted average of those rates, where the weights are driven by how much time a gene tree branch spends in each species tree branch.

Alignment simulations: On these scaled genealogies, we simulate mutations under the GTR model with a mean substitution rate of 1.909 per bp per generation, calculated based on the avian species tree in substitution units. We introduce mutation-rate heterogeneity both across genomic regions and across sites. Specifically, *i*) we partition the genome into random contiguous segments with a mean length of 10^4 bp and scale the substitution rate of each segment by a random multiplier drawn from a high-variance Gamma distribution. *ii*) Within each segment, we further scale the mutation rate at each site using a Gamma distribution with 20-fold lower variance. The (inverse) variance parameter itself is drawn from a log-normal distribution independently for each independent block.

Gene tree estimations: We sample 500bp genes from each 4Kbp window, respecting the concatenation boundaries, and infer gene trees that we use in our benchmarking using IQTREE (v2.4.0) and GTR+G4 model with `--abayes` option enabled.
